# Supplementary material for: Inter-membrane association of the Sec and BAM translocons for bacterial outer-membrane biogenesis
Source: eLife. 2020 Nov 4;9:e60669. doi: 10.7554/eLife.60669 (PMC7695460; doi:10.7554/eLife.60669)
Supplement: Supplementary file 1 [file elife-60669-fig2-data1.odt]

Table 2: Image processing details

|  | Complex | Initial particles | High quality particles | Final 3D volume | Resolution |
| --- | --- | --- | --- | --- | --- |
| HTL structures | HTL-DDM | 100754 | 74070 | - | - |
| HTL-DDM+CL | 22869 | 18674 | - | - |
| HTLafter gradient+CL | 21735 | 18434 | - | - |
| HTLafter gradient+gluta.+CL | 101142 | 90432 | 9656 (compact conformation) | 26,56 Å |
| 1569 (extended conformation) | 39,54 Å |
| HTL519D519N | 42098 | 38052 | 20699 (compact conformation) | 18,7 Å |
| 4451 (extended conformation) | 20,70 Å |
| HTL-BAM structures | HTL:BAM Δgluta | 29336 | 12460 | - | - |
| HTL:BAM grafix | 65381 | 28646 | 5122 | 37,2 Å |
| HTL:BAM grafix ΔCL | 14530 | 11406 | - | - |
| HTL:BAM CryoEM | 839845 | 608493 | 12779 | 18,23 Å |
| SecDF:BAM | 22642 | 11987 | 5430 | 39,4 Å |
| HTLΔYidC:BAM | 16008 | 8612 | 4943 | 36,7 Å |
| HTL:BAMΔB | 31561 | 11484 | 3919 | 33,65 Å |
